# Assessment value of interleukin‐6, procalcitonin, and C‐reactive protein early kinetics for initial antibiotic efficacy in patients with febrile neutropenia: A prospective study

**DOI:** 10.1002/cam4.7307

**Published:** 2024-07-05

**Authors:** Haifeng Zheng, Zimian Luo, Yafei Yi, Kang Liu, Zhongjun Huo, Yaqin You, Hujiao Li, Min Tang

**Affiliations:** ^1^ Department of Hematology Central Hospital of Xiangtan Xiangtan China; ^2^ Department of Hematology Changsha Central Hospital Changsha China

**Keywords:** C‐reactive protein, febrile neutropenia, initial antibiotic efficacy, interleukin‐6, procalcitonin

## Abstract

**Background:**

This study aims to investigate the early kinetics of interleukin 6 (IL‐6), procalcitonin (PCT), and C‐reactive protein (CRP) on initial antibiotic efficacy in hematological disorder patients with febrile neutropenia (FN).

**Methods:**

A total of 40 patients with 43 episodes of FN were enrolled and divided into initial antibiotic effective group (IAE group, *n* = 24) and initial antibiotic ineffective group (IAI group, *n* = 19). The levels of IL‐6, PCT, and CRP before antibacterial treatment (T0), and 12 h (T1), 24 h (T2), 48 h (T3), and 72 h (T4) post‐antibacterial treatment were determined, respectively. Furthermore, the receiver operating characteristic curve (ROC) analysis was performed to evaluate the clinical value of indicators.

**Results:**

In IAE group, the IL‐6 levels gradually decreased from T0 to T4, and the CRP levels significantly decreased at 48 to 72 h, whereas both IL‐6 and CRP remained at high levels in the IAI group. The PCT levels in both groups increased at the early stage of anti‐infection (T1–T2) and reached to peak at T1–T2 in effective group. ROC curve analysis identified IL‐6 as a predictive biomarker for initial antibiotic efficacy at 12, 48, and 72 h after treatment, with the AUC of 0.698, 0.744, and 0.821, respectively. In addition, CRP demonstrated predictive ability of initial antibiotics against infection at 24, 48, and 72 h after therapy, with the AUC of 0.724, 0.741, and 0.797, respectively. ROC curve analysis of percentage changes demonstrated that IL‐6 percentage change showed predictive ability of antibiotic efficacy at the early stage, and both the IL‐6 and CRP percentage changes showed the predictive ability of antibiotic efficacy 48 or 72 h after antibiotics therapy.

**Conclusion:**

This study confirmed IL‐6 and CRP levels, and the percentage change in IL‐6 as the biomarkers for initial antibiotic efficacy prediction in hematological disorder patients with FN.

## 1 INTRODUCTION

Neutrophil deficiency is a common adverse reaction in tumor patients treated with chemotherapy, leading to host extremely vulnerable to numerous microorganisms, and even a fatal infection.^1^, ^2^ Febrile neutropenia (FN) is considered as a medical emergency due to the inflammatory response is suppressed and fever is often the only symptom of infection.^3^, ^4^ In recent 5 decades, despite the significant advance in FN management due to the development of empirical antibiotic regimens,^4^ inappropriate initial antibiotic therapy may occur,^5^ which may be associated with differences in local microbial epidemiology and the emergence of multidrug resistant (MRD) Gram‐negative bacteria.^6^ Sufficient evidences indicated the early use of ineffective antibiotics against infection has a strong adverse effect on the survival rate, and the incidence of serious complications and mortality of patients with initial antibiotic insensitive are significantly higher than that of the initial antibiotic sensitive patients.^7^ In FN, once inappropriate antibiotics are started, it usually takes several days to correct.^1^, ^5^

At present, there are no immune response biomarkers that can directly measure the intrinsic pathogen load of the host.^8^ It has been realized that in the context of antibiotic treatment, observing the kinetic characteristics and dynamic changes in biomarkers during anti‐infection may explain the efficacy of antimicrobial therapy.^5^, ^9^ Under infection, interleukin 6 (IL‐6) usually appears in plasma at 30 min to 2 h after stimulation, reaches its peak at 4–6 h, and has a half‐life of about 4–6 h; Procalcitonin (PCT) usually appeared in plasma 2 to 3 h after stimulation, reached its peak at 12 to 24 h after infection, and its half‐life was about 20 to 28 h; C‐reactive protein (CRP), however, often appears in plasma at 6 to 8 h after stimulation, reaches its peak at 24 to 48 h, and has a half‐life of 19 h. For decades, interleukin 6 (IL‐6), procalcitonin (PCT), and C‐reactive protein (CRP) have been widely investigated to identify pathogenic bacteria, evaluate the severity of infection, and predict adverse outcomes in cancer patients.^10^, ^11^, ^12^, ^13^, ^14^ However, the function of IL‐6, PCT and CRP in predicting antibiotic efficacy is far from clear, especially in FN patients.^15^ Herein, we conducted a prospective study to investigate whether IL‐6, PCT, and CRP can be used to evaluate initial antibiotic efficacy of FN patients with hematological disorder through observing the early kinetic changes in these three biomarkers.

## 2 MATERIALS AND METHODS

### 2.1 Study design

This is a prospective and observational study at Xiangtan Central Hospital investigating the evaluation role of IL‐6, PCT, and CRP in initial antibacterial efficacy of FN patients. This study was approved by the Ethics Committee of Xiangtan Central Hospital (Ethics Approval Letter No. 2021‐10‐002), and eligible patients provided written informed consent.

Adults FN patients suspected of bacterial infection or bacterial combined with fungal infection and initiated empirical antimicrobial therapy were enrolled in this study. When the interval was more than 2 weeks, FN was considered as an independent episode, requiring the patient to perform clinically well during both FN. All enrolled patients had clear underlying diseases, and exclude patients with fever caused by the primary disease, who are younger than 18 years old, who have received antibiotic treatment within 48 h, who received medications that can suppress or alter the inflammatory response, such as rituximab or steroids, 3 days before the fever, with the conditions of interfering inflammatory response, such as acute renal replacement therapy and cardiopulmonary resuscitation within the first 24 h, with fungal or/and viral infections, or with fever caused by non‐bacterial infections such as hemophagocytic syndrome‐, neoplasm‐, and drug‐induced fever. After enrollment, patient clinical data, treatment records, pathogen laboratory test results, and antibiotic effective/ineffective were collected for three consecutive days. The levels of peripheral blood IL‐6, PCT, and CRP before antibiotics treatment were served as the baselines (T0). Approximately 5 mL of peripheral blood samples (Divided into thirds) were then continuously collected after 12 h (T1), 24 h (T2), 48 h (T3), and 72 h (T4) of anti‐infection therapy to evaluate the concentrations of IL‐6, PCT, and CRP. The samples were centrifuged within 4 h, and the upper serum was collected and maintained at −80°C. All specimens were tested uniformly after thawing. Initial empirical therapy with antibiotics was performed according to the Guidelines for Clinical Application of Antibiotics in Patients with Fever and Lack of Neutrophils in China (2020). The antibiotic efficacy was evaluated as effective and ineffective according to the Guidelines for Clinical Application of Antimicrobial Drugs (2015), and the patients were divided into initial antibiotic effective group (IAE group) and initial antibiotic ineffective group (IAI group) accordingly. All the antibiotic efficacy assessment was judged by at least two physicians.

### 2.2 Definition

1. FN: Absolute neutrophil count (ANC) <0.5 × 10^9^/L or expected decrease to <0.5 × 10^9^/L within 48 h; axillary temperature >38.3°C once or >38°C twice, lasting at least 1 h or 2 measurements within 12 h.
2. Fever classifications were defined according to the International Society of Immunodeficiency Hosts.

Bacteremia (MDI): Defined as fever with positive blood culture bacteria (peripheral blood or central venous catheter), accompanied with or without sepsis; Microbiological evidence of infection based on positive blood, urine, stool, throat, bronchoalveolar lavage, cerebrospinal fluid, or wound culture, classified as bacteremia (MDI‐B);

Clinical evidence of infection: The manifestations of infection were consistent with radiological findings;

Fever of unknown origin: Fever without observation of clinical infection site or microbiological infection.

### 2.3 Detection of IL‐6, PCT, and CRP

The serum concentration of IL‐6 was measured by the multiplex microsphere flow immunofluorescence luminescence method (RAISECARE, Qingdao, China). The intra‐analytical coefficient of variation (CV) was ≤15% with a linear range from 2.44 to 10,000 pg/mL. The PCT concentration was determined by immunofluorescence dry quantitation method using PCT measurement reagent on a Jet‐istar 3000 immunoassay analyzer (John Shengtai Biotechnology Co., Ltd., Hangzhou, China). The CV was ≤15%, and the detection range is between 0.1 and 100 ng/mL. As for CRP detection, the immunoturbidimetry was performed using a cobas c 702 Roche Chemistry analyzer (Roche Diagnostics Switzerland). The CV was <20% with a detection range from 0.3 to 350 mg/L.

The percentage change of IL‐6, PCT, or CRP in patients across time periods was calculated by the following formula: (Tn‐T0)/T0 × 100%. Tn represents T1, T2, T3, or T4.

### 2.4 Statistical analysis

Data statistics were analyzed using the IBM SPSS (version 26.0). Enumeration data were expressed as frequency (n) and percentage (%). Measurement data were described as median (interquartile range, IQR) and tested using the Mann–Whitney *U*‐test in effective group and ineffective group and using the Friedman ANOVA in antibacterial period. Receiver operating characteristic (ROC) curve analysis was performed to evaluate the predictive ability of IL‐6, PCT, and CRP absolute concentrations and percentage change against infection efficacy, and the optimal cutoff values were obtained based on Youden index (sensitivity + specificity‐1). *p* < 0.05 indicates a statistical significance.

## 3 RESULTS

### 3.1 Baseline characteristics

This study recruited a total of 40 patients with 43 episodes of FN collected. Among the 43 infectious fevers, 7 had bacteremia infection, 24 had pulmonary infection, 4 had skin soft tissue infection, 3 had urinary tract infection, 1 had perianal infection, 1 had intestinal infection, and 11 had unexplained infection. Partial patients had multiple sites of infection, such as pulmonary infection combined with skin soft tissue infection, and the following causative microorganisms were cultured in 43 episodes of infectious fever: *Escherichia coli*, *Staphylococcus hominis*, *Klebsiella pneumoniae subsp. Pneumoniae*, and *Staphylococcus epidermidis*. In addition, among the 43 infectious fevers, initial antibiotics were effective in 24 patients (55.81%), whereas 19 patients (44.19%) were ineffective (antibiotics were changed in time). Patients clinical baseline characteristics are shown in Table 1, demonstrating only the duration of fever and the incidence of hepatic dysfunction were statistically significant between IAE and IAI groups. Four patients in IAI group changed antibiotics between 48 and 72 h, and the kinetics IL‐6, PCT, and CRP of showed no significant impact compared with the other patients (data not shown).

**TABLE 1:** Baseline characteristics comparison between antibiotic effective and ineffective groups.

	Effective (*n* = 24)	Ineffective (*n* = 19)	*p*‐value
Age, year			
Median (interquartile range)	56 (48–71.5)	52 (38–62)	0.445
Gender, male/female	15/9	8/11	0.228
Duration of the fever, day			
Median (interquartile range)	3.5 (2–5.75)	8 (5–11)	0.002*
Duration of neutrophil deficiency, day			
Median (interquartile range)	7 (3–10)	9 (5–16)	0.058
Number of neutrophils at fever, 10^9^/L			
Median (interquartile range)	0.28 (0.085–0.585)	0.11 (0.02–0.75)	0.583
Utilization of neutrophil‐stimulating factor			
Yes/no	15/9	13/6	0.775
Disease type, *n* (%)			0.222
Lymphadenoma	5 (20.83)	2 (10.53)	
Acute leukemia	8 (33.33)	13 (68.42)	
Myelodysplastic syndrome	4 (16.67)	2 (10.53)	
Multiple myeloma	3 (12.50)	0 (0.00)	
Others	4 (16.67)	2 (10.53)	
Causes of fever, *n* (%)			0.910
Bacteremia	4 (16.67)	3 (15.79)	
Clinically recorded infection	13 (54.17)	12 (63.16)	
Unknown	7 (29.17)	4 (21.05)	
Initial antibiotic type, *n* (%)			0.729
β‐lactamase inhibitor compound	8 (33.33)	6 (3.58)	
Third generation cephalosporin	7 (29.17)	7 (36.84)	
Carbapenems	8 (33.33)	4 (21.05)	
Others	1 (4.17)	2 (10.53)	
Complication, yes/no			
Hepatic insufficiency	7/17	0/19	0.012*
Renal insufficiency	3/21	2/17	1.000
Fungal infection	4/20	1/19	0.363

* *p* < 0.05.

### 3.2 Kinetic changes of IL‐6, PCT, and CRP

**FIGURE 1:**
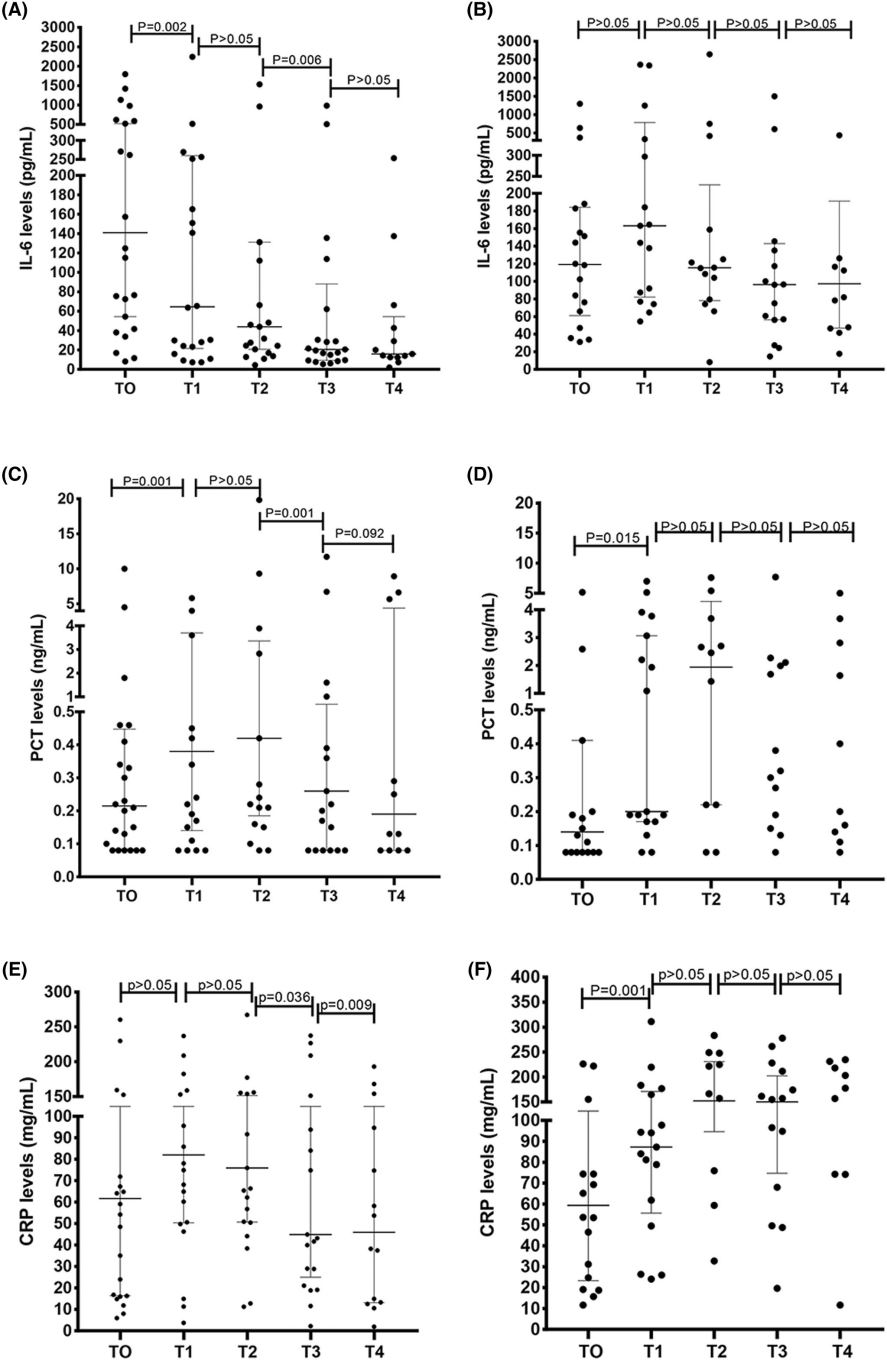
Kinetic changes in IL‐6, PCT, and CRP. (A) Kinetic changes of IL‐6 in the initial antibiotic therapy effective group. (B) Kinetic changes of IL‐6 in the initial antibiotic therapy ineffective group. (C) Kinetic changes of PCT in the initial antibiotic therapy effective group. (D) Kinetic changes of PCT in the initial antibiotic therapy ineffective group. (E) Kinetic changes of CRP in the initial antibiotic therapy effective group. (F) Kinetic changes of CRP in the initial antibiotic therapy ineffective group.

As shown in Figure 1A, the IL‐6 level in the IAE group gradually decreased from T0 to T4, and the median (interquartile range) were 141.04 (44.83–570.37), 64.54 (21.50–259.76), 43.85 (18.82–232.59), 20.59 (9.36–88.00), and 15.96 (12.39–54.43), respectively. At the early stage of anti‐infection (T1), majority of patients (91.30%, 21/23) in the IAE group showed decreased IL‐6 level compared with the baseline concentration (*p* = 0.002). However, in the IAI group, IL‐6 was consistently at high levels during the anti‐infection process without any significant different (Figure 1B, all *p* > 0.05). The PCT levels in both effective and ineffective groups increased at the early stage of anti‐infection (T1–T2) (Figure 1C,D) and reached to peak at T1–T2 in effective group. Majority of patients (73.68%, 14/19) in the IAE group showed decreased PCT concentrations at T3 (P = 0.001) and maintained low level at T4. However, in the IAI group, PCT was consistently at high levels at T3 and T4. The peak of PCT in the IAI group was delayed compared with that in effective group. As for CRP, in the IAE group, CRP levels showed no statistic difference in the early anti‐infection (12–24 h) compared with baseline, and significantly decreased at 48 to 72 h (T3–T4) (Figure 1E). In IAI group, CRP levels elevated at the early stage of anti‐infection (T1) and remained at high level throughout the subsequent anti‐infection courses (24–72 h) (Figure 1F). The detail levels of IL‐6, PCT, and CRP at different stage in both groups are shown in Table 2.

**TABLE 2:** Levels of IL‐6, PCT, and CRP at different time points in the effective and ineffective groups.

Biomarkers	Group	T0	T1	T2	T3	T4
IL‐6	Effective	141.04 (44.83–570.37)	64.54* (21.50–259.76)	43.85 (18.82–232.59)	20.59* (9.36–88.00)	15.96 (12.39–54.43)
	Ineffective	119.21 (61.10–184.25)	163.12^#^ (82.14‐789.19)	115.35^#^ (78.13‐224.26)	96.34^#^ (56.34–142.90)	97.195^#^ (46.80–191.14)
PCT	Effective	0.22 (0.09–0.45)	0.38* (0.14–3.70)	0.42 (0.19–3.36)	0.26* (0.08–0.84)	0.19 (0.08–4.39)
	Ineffective	0.14 (0.08–0.44)	0.20* (0.17–3.42)	1.95 (0.22–3.92)	0.53 (0.21–2.23)	0.84 (0.15–2.23)
CRP	Effective	61.63 (16.40–130.09)	81.97* (49.75–146.25)	75.94 (50.70–151.38)	44.94* (24.97–136.18)	45.97* (13.10–109.50)
	Ineffective	59.37* (23.30–124.65)	87.26* (55.65–170.88)	151.94^#^ (94.62–230.80)	150.29^#^ (74.69–202.29)	143.63^#^ (99.76–210.65)

*Note*: Data are presented as median (interquartile range).

* *p* < 0.05, Labeled period versus adjacent period (before);

^#^ *p* < 0.05, effective group versus ineffective group.

### 3.3 Evaluation the predictive ability of IL‐6, PCT, and CRP levels against infection efficacy

**FIGURE 2:**
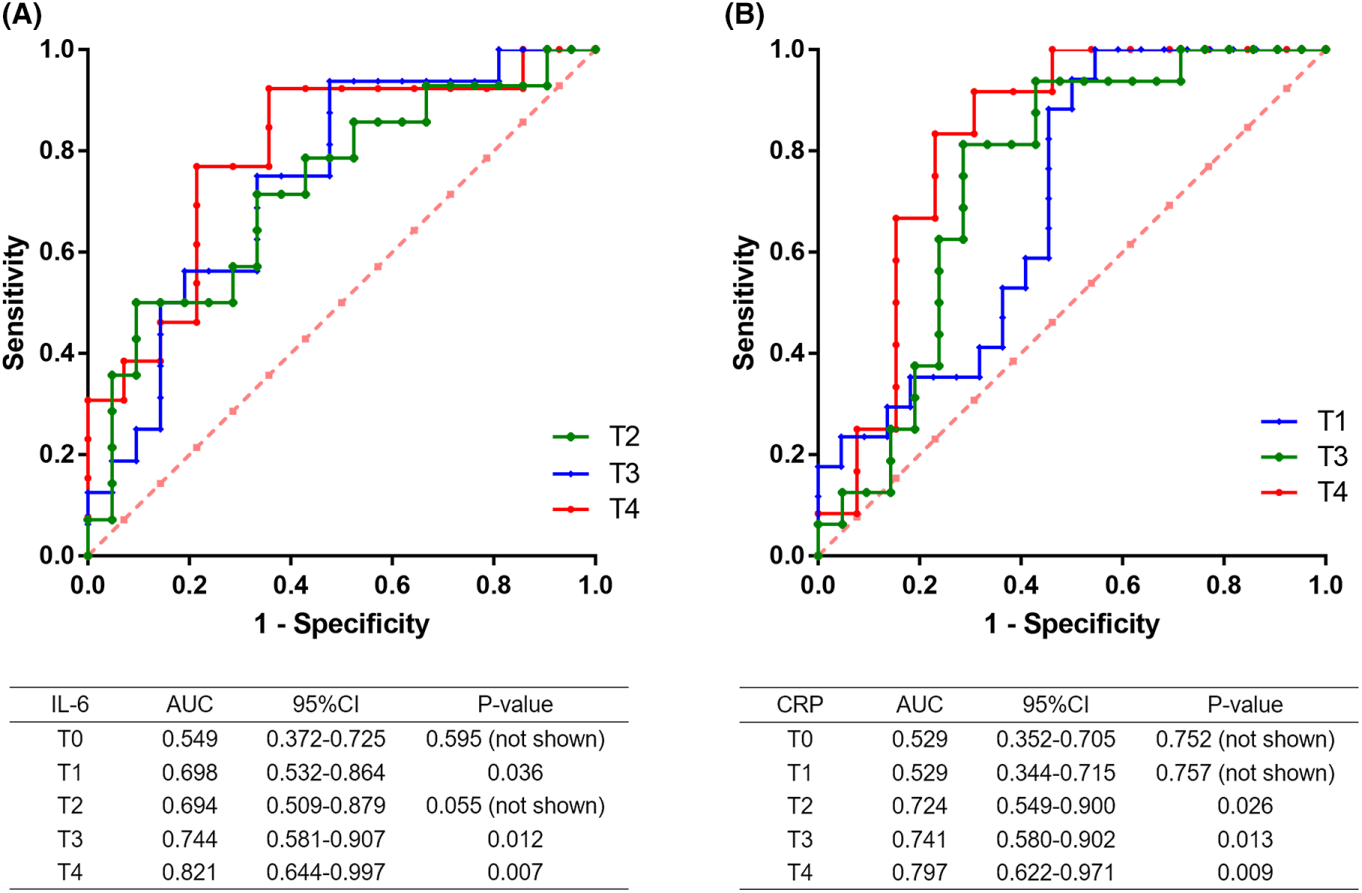
Evaluation the predictive ability of IL‐6 and CRP levels against infection efficacy. (A) The predictive ability of IL‐6 levels against infection efficacy was evaluated using ROC curves. (B) The predictive ability of CRP levels against infection efficacy was evaluated using ROC curves.

ROC curve analysis was performed to evaluate the predictive ability of IL‐6, PCT, and CRP absolute concentrations against infection efficacy. As shown in Figure 2A, IL‐6 was found to be predictive in assessing the efficacy of initial antibiotic anti‐infection at 12 h (T1), 48 h (T3), and 72 h (T4), with the optimal cutoff value of 42.52 pg/mL (area under the ROC curve (AUC) = 0.698, 95% confidence interval (CI) 0.532–0.8640, sensitivity 100%, and specificity 54.5%), 43.35 pg/mL (AUC = 0.744 95%CI 0.581–0.907, sensitivity 81.3%, specificity 71.4%) and 35.31 pg/mL (AUC = 0.821, 95% CI 0.644–0.997, sensitivity 91.7%, and specificity 69.2%), respectively, indicating the predictive assessment ability of IL‐6 increased with the duration of resistance to infection. CRP demonstrated predictive ability of initial antibiotics against infection at 24 h (T2), 48 h (T3), and 72 h (T4), with the optimal cutoff value of 156.6 mg/L (AUC = 0.724, 95% CI 0.549–0.900, sensitivity 50%, and specificity 90.5%), 46.87 mg/L (AUC = 0.741, 95% CI 0.580–0.902, sensitivity 93.8%, and specificity 52.4%), and 66.21 mg/L (AUC = 0.797, 95% CI 0.622–0.971, sensitivity 92.3%, and specificity 64.3%), respectively (Figure 2B). However, serum PCT level cannot be used to predict the anti‐infection efficacy (data not shown).

### 3.4 Evaluation the predictive ability of IL‐6, PCT, or CRP percentage change in patients across time periods against infection efficacy

**FIGURE 3:**
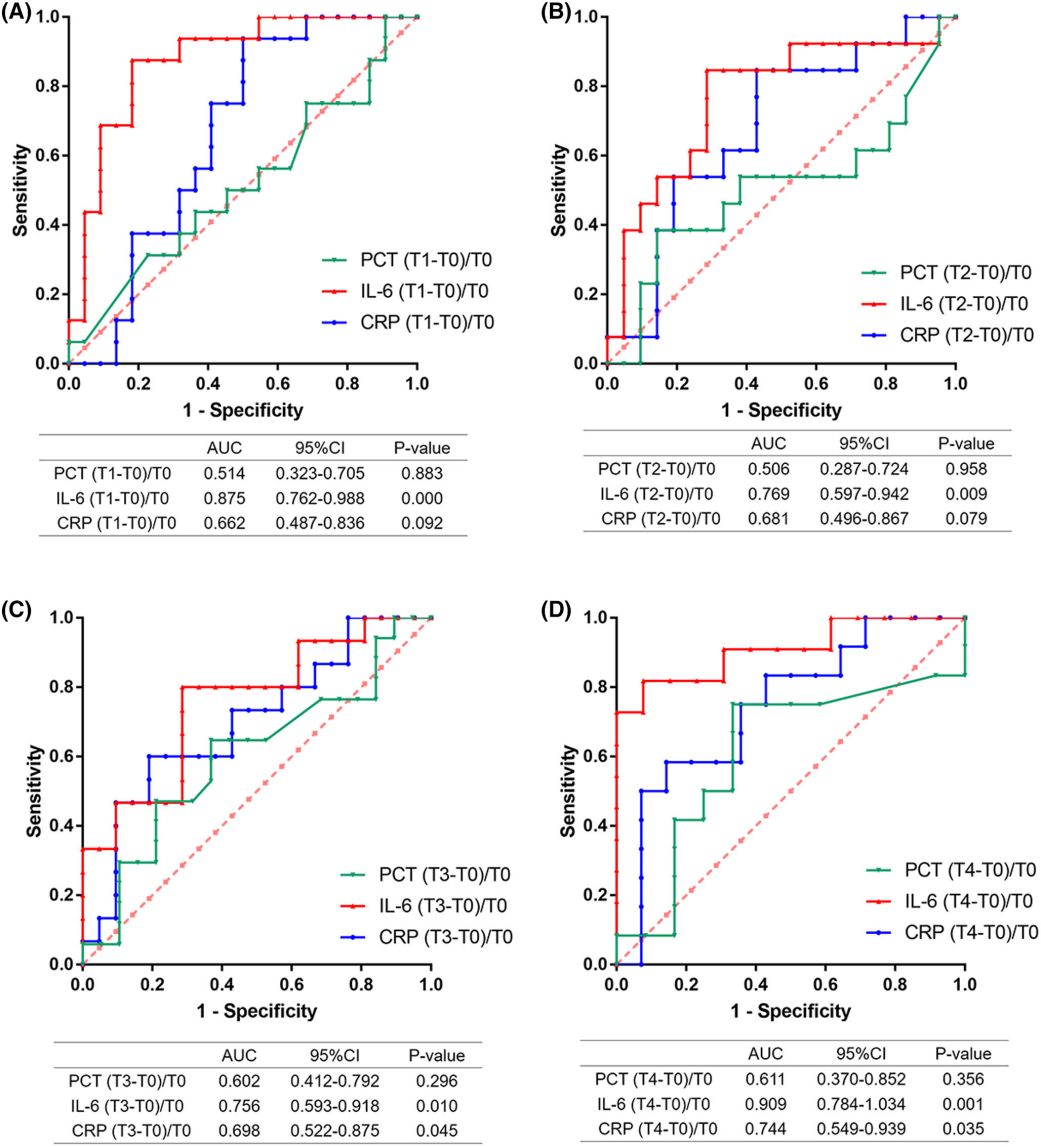
Evaluation the predictive ability of IL‐6, PCT or CRP percentage change in patients across time periods against infection efficacy. (A) The predictive ability of IL‐6, PCT, or CRP percentage change at 12 h in patients against infection efficacy was evaluated using ROC curves. (B) The predictive ability of IL‐6, PCT, or CRP percentage change at 24 h in patients against infection efficacy was evaluated using ROC curves. (C) The predictive ability of IL‐6, PCT, or CRP percentage change at 48 h in patients against infection efficacy was evaluated using ROC curves. (D) The predictive ability of IL‐6, PCT, or CRP percentage change at 72 h in patients against infection efficacy was evaluated using ROC curves.

Furthermore, ROC curve analysis was conducted to evaluate the predictive ability of IL‐6, PCT, or CRP percentage change in patients across time periods against infection efficacy, demonstrating that only IL‐6 percentage change showed predictive ability of anti‐infection efficacy at the early stage (T1–T2) (Figure 3A,B). Youden index analysis revealed the optimal cutoff value of IL‐6 percentage change is −11.85% (AUC = 0.875, 95% CI 0.762–0.988, sensitivity 87.5%, and specificity 81.8%) at T1 and −36.24% (AUC = 0.769, 95% CI 0.597–0.942, sensitivity 84.6%, and specificity 71.4%) at T2, suggesting that the level of IL‐6 decreases by 11.85% at T1 or 36.24% at T2 compared with baseline may demonstrate the failure of anti‐infective therapy. In addition, 48 or 72 h after antibiotics therapy, both the IL‐6 and CRP percentage changes showed the predictive ability of anti‐infection efficacy (Figure 3C,D). Whereas, the percentage change of serum PCT cannot be used to predict the anti‐infection efficacy at the early stage.

## 4 DISCUSSION

It is complex for clinical decision of antibiotic therapy, especially FN due to its absence of typical clinical symptoms.^1^ During treatment, serial examinations are usually required to assess response of treatment to ensure a favorable prognosis.^8^ Considering the insignificant leukocyte changes and imaging delay in patients with FN, as well as body temperature is affected by many factors,^5^ objective indicators are needed to predict and evaluate anti‐infection efficacy of FN at the early stage.

Previous studies have demonstrated the significant decrease in IL‐6 on the third day (48–72 h) of treatment in patients with initial antibiotic efficacy.^16^, ^17^ In this study, we advanced the observation time to 12 h, finding that unlike IL‐6 was consistently at high levels in the IAI group, IL‐6 reduced obviously at the early stage of anti‐infection (12 h) in the IAE group, which is similar to the study reported by Delgado et al., revealing that IL‐6 reached to a peak value at the onset of fever, followed by a decrease on the second day (12–24 h).^15^ This similarity may be due to the low likelihood of complications in patients who respond quickly to initial antibiotics. Further analysis using the ROC curve found that the AUC of IL‐6 to distinguish the effectiveness of anti‐infection at 12 h was 0.895, whereas PCT and CRP were failed to predict the antimicrobial efficacy, which may be associated with the unique characteristics of these three biomarkers. IL‐6 usually appears in plasma 30 min to 2 h after stimulation, reaching its peak at 4–6 h, with a half‐life of approximately 4–6 h^18^; PCT often appears in plasma 2–3 h after stimulation, reaching its peak at 12–24 h after infection, with a half‐life of approximately 20–28 h^19^; While CRP often appears in plasma 6–8 h after stimulation, reaching its peak at 24–48, with a half‐life of 19 h.^19^ This means that compared with PCT and CRP, IL‐6 is a fast biomarker. Unlike most clinical studies considering threshold level to guide clinical decisions, our present study examined the kinetic changes of biomarkers to evaluate the initial antibacterial efficacy of patients with FN, suggesting that IL‐6 not only predicts antibiotic efficacy at the early stage but also has significantly better predictive ability than PCT and CRP.

Antibiotic suitability prediction of PCT against infection at early stage has been reported in patients with sepsis. Trásy et al., have observed the kinetic changes of PCT per 8 h within 24 h prior to anti‐infection treatment, finding that an increase in PCT concentration of >69.2% in the first 16 h or an increase of >73.5% within 24 h suggests inappropriate antibiotic treatment.^5^ Studies that evaluated initial antibiotic efficacy in FN patients through observing the change of PCT level have also been conducted, demonstrating the familiar of peak value (0.5 ng/mL) delay in patients receiving inappropriate antimicrobial therapy.^17^, ^20^ Further study by Leticia et al. has found that the positive predictive value reached 83% when the level of PCT is more than 4.8 ng/mL on the third day of anti‐infection, suggesting the inappropriate antibiotic therapy.^21^ Our study also showed a peak delay in the ineffective group. This may be related to the biological characteristics of procalcitonin. PCT usually appears in plasma 2 to 3 h after stimulation, reaches its peak at 12 to 24 h after infection, and has a half‐life of about 20 to 28 h. In the initial of infection, if antibiotics administered during the early stage are ineffective, the inflammatory response may escalate progressively, inducing continued procalcitonin production. However, unlike the research of Leticia et al,^21^ we found little significance of PCT level or percentage change to evaluate anti‐infection efficacy, which may be related to infection severity or leukopenia. In contrast to Leticia's study, where up to 29% of the population died, most individuals in our study showed good outcomes, with only one person experiencing septic shock and one person eventually dying. Svaldi et al. revealed that PCT levels never reached 2 ng/mL in the case of severe leukopenia, even in the patients with severe sepsis or septic shock.^22^ Additionally, the concentration of PCT was reduced to 1/2–1/3 of the normal value in patients with severe FN (absolute neutrophil count <0.1 × 10^9^/L). In our present study, 34.28% (12/35) of patients experienced severe FN, and 31.43% (11/35) of patients did not detect PCT (0.1 ng/mL) during fever. Moreover, evidence has suggested that the elevation of PCT in patients with FN often occurs at the late stage of infection, mainly due to multiple organ failure.^23^ Collectively, given the possible reduction of PCT in patients with FN, it may have little significance in predicting and assessing the initial antibiotic efficacy in patients with FN.

Whether CRP can guide clinical utilization of antibiotic in patients with FN is currently far from clear. Previous study has delineated that CRP remains at high level during anti‐infection process and cannot predict antimicrobial efficacy.^17^ However, in our study, the concentration of CRP remained at high level in the IAI group, but decreased significantly 24 h after anti‐infection in IAE group, which is similar to previous studies, demonstrating that CRP rapidly decreased after reaching its peak in anti‐infection effective group.^8^, ^15^ This difference may be associated with the low specificity of CRP, which is not only produced in infected patients, but affected by the disease severity in patients. In this study, the distinguished kinetic of CRP in patients with different therapeutic efficacy may be due to the fact that the enrolled population is largely non‐critical patients, and the CRP changes are only affected by the infectious pathogens. In addition, it has been reported that proliferative diseases of the hematopoietic system show an impact on baseline CRP, such as lymphoma induces significantly higher initial CRP levels, while leukemia causes moderate increase or no affect at all,^11^ which can be ignored to a certain degree in this study due to the percentage change in CRP was used rather than a specific cutoff value.

Furthermore, we used the baseline levels of these three biomarkers to predict the source of infection, indicating that despite the levels IL‐6 and PCT were higher in bacteremia than that in clinical records, no statistical difference was observed. This may be related to the small number of bacteremia (7 cases) in our study. It is controversial of IL‐6 clinical in bacteremia patients with FN. Evidence has confirmed that IL‐6 cannot predict but can exclude bacteremia due to the low sensitivity and PPV values. However, Diepold et al. have found that IL‐6 is the best predictor of bacteremia and severe bacterial infection, with high sensitivity and specificity (90% and 85%, respectively).^24^ As for PCT, Lai et al. have found the lowest AUC of bacteremia in patients with FN compared with other subgroups (A total of 50,933 patients with bacteremia were included).^25^ Further study of FN patients (A total of 1466 blood samples were included) reported by Luo et al. obtained a similar conclusion that the AUC of PCT distinguishing bacteremia in patients with FN was 0.685,^26^ indicating PCT as an invalid biomarker for bacteremia diagnosis. Kitanovski et al. have measured the concentration of CRP on two consecutive days in 90 FN episodes experienced by 47 children, finding that although the concentration of CRP in patients with bacteremia is significantly higher than that in non‐bacteremia patients, the diagnostic accuracy is unsatisfied,^27^ which is consistent with the conclusion of Doerflinge and de Araujo demonstrating CRP shows a low value for bacteremia diagnosis.^7^, ^28^ In addition to its slow kinetics, this may also be associated with the influence of malignant disease and tissue damage on CRP levels.^27^

This study has several limitations. First, this is a single‐center study with small sample size. In addition, the various of disease type limits the universality of the results, although most individuals are tumor patients. Second, this is an observational cohort and the use of antibiotics depends on the judgment of physician. Third, although majority of the individuals were hematological tumor patients, no further stratification of the results based on basic diagnosis or chemotherapy regimen due to the limited cases.

## 5 CONCLUSION

In conclusion, we currently observe the kinetic changes of IL‐6, PCT, and CRP to investigate their role in initial antibiotic efficacy evaluation of FN patients with hematological disorder, confirming IL‐6 and CRP as the biomarkers. Within 72 h of anti‐infection, the predictive ability of serum IL‐6 and CRP increases with the prolongation of antibiotic time. In addition, the percentage change in IL‐6 shows promising application prospects to predict anti‐infection efficacy within 12 h of antibiotic therapy. However, an expanded and multiple centers cohort is required to complement and strengthen our conclusion.

## AUTHOR CONTRIBUTIONS

**Haifeng Zheng:** Conceptualization (equal); data curation (equal); formal analysis (equal); validation (equal); visualization (equal); writing – original draft (equal). **Zimian Luo:** Conceptualization (equal); methodology (equal); supervision (equal); writing – review and editing (equal). **Yafei Yi:** Data curation (equal); methodology (equal); validation (equal). **Kang Liu:** Data curation (equal); methodology (equal); validation (equal). **Zhongjun Huo:** Data curation (equal); methodology (equal); validation (equal). **Yaqin You:** Data curation (equal); methodology (equal); validation (equal). **Hujiao Li:** Data curation (equal); methodology (equal); validation (equal). **Min Tang:** Data curation (equal); methodology (equal); validation (equal).

## CONFLICT OF INTEREST STATEMENT

The authors have no potential conflict of interest to declare.

## ETHICS STATEMENT

This study was approved by the Medical Ethics Committee of Xiangtan Central Hospital (Approval number: 2021‐10‐002).

## Data Availability

The data that support the findings of this study are available from the corresponding author upon reasonable request.
